# Evaluation of Selected Plant Phenolics via Beta-Secretase-1 Inhibition, Molecular Docking, and Gene Expression Related to Alzheimer’s Disease

**DOI:** 10.3390/ph17111441

**Published:** 2024-10-28

**Authors:** Tugba Uçar Akyürek, Ilkay Erdogan Orhan, F. Sezer Şenol Deniz, Gokcen Eren, Busra Acar, Alaattin Sen

**Affiliations:** 1 Department of Pharmacognosy, Faculty of Pharmacy, Gazi University, 06330 Ankara, Türkiye; tugba.ucar@tarimorman.gov.tr (T.U.A.); fssenol@gazi.edu.tr (F.S.Ş.D.); 2 Department of Pharmacognosy, Faculty of Pharmacy, Lokman Hekim University, 06510 Ankara, Türkiye; 3 Department of Pharmaceutical Chemistry, Faculty of Pharmacy, Gazi University, 06330 Ankara, Türkiye; gokcene@gazi.edu.tr; 4 Department of Molecular Biology & Genetics, Faculty of Life & Natural Sciences, Abdullah Gül University, 38080 Kayseri, Türkiye; busra.acar@agu.edu.tr (B.A.); sena@agu.edu.tr (A.S.); 5 Department of Biology, Faculty of Science, Pamukkale University, 20070 Denizli, Türkiye

**Keywords:** Alzheimer’s disease, beta secretase, cytotoxicity, gene expression, molecular docking, neuroprotection, phenolic compounds

## Abstract

**Background:** The goal of the current study was to investigate the inhibitory activity of six phenolic compounds, i.e., rosmarinic acid, gallic acid, oleuropein, epigallocatechin gallate (EGCG), 3-hydroxytyrosol, and quercetin, against β-site amyloid precursor protein cleaving enzyme-1 (BACE1), also known as β-secretase or memapsin 2, which is implicated in the pathogenesis of Alzheimer’s disease (AD). **Methods and Results:** The inhibitory potential against BACE1, molecular docking simulations, as well as neurotoxicity and the effect on the AD-related gene expression of the selected phenolics were tested. BACE1 inhibitory activity was carried out using the ELISA microplate assay via fluorescence resonance energy transfer (FRET) technology. Molecular docking experiments were performed in the human BACE1 active site (PDB code: 2WJO). Neurotoxicity of the compounds was carried out in SH-SY5Y, a human neuroblastoma cell line, by the Alamar Blue method. A gene expression analysis of the compounds on fourteen genes linked to AD was conducted using the real-time polymerase chain reaction (RT-PCR) method. Rosmarinic acid, EGCG, oleuropein, and quercetin (also used as the reference) were able to inhibit BACE1 with their respective IC_50_ values 4.06 ± 0.68, 1.62 ± 0.12, 9.87 ± 1.01, and 3.16 ± 0.30 mM. The inhibitory compounds were observed to occupy the non-catalytic site of the BACE1. However, hydrogen bonds were found to be present between rosmarinic acid and EGCG and aspartic amino acid D228 in the catalytic site. Oleuropein and quercetin effectively suppressed the expression of *PSEN*, *APOE*, and *CLU*, which are recognized to be linked to the pathogenesis of AD. **Conclusions:** The outcomes of the work bring quercetin, EGCG, and rosmarinic acid to the forefront as promising BACE1 inhibitors.

## 1. Introduction

Alzheimer’s disease (AD) is a neurodegenerative disorder characterized by the accumulation of amyloid-β (Aβ) plaques and tau protein tangles in the brain, leading to progressive cognitive decline and memory loss [1]. While the exact cause of AD remains elusive, several pathological mechanisms have been proposed and widely studied. The amyloid cascade hypothesis posits that the accumulation and aggregation of Aβ peptides, particularly Aβ_42_, play a central role in the pathogenesis of AD. Aβ peptides aggregate to form insoluble plaques, which are neurotoxic and disrupt synaptic function, leading to neuronal dysfunction and death. The other pathological mechanism for AD is the tau hypothesis, which proposes that abnormal phosphorylation and aggregation of tau protein contribute to the development and progression of AD [2]. Tau protein is a microtubule-associated protein involved in stabilizing neuronal microtubules. Moreover, synaptic dysfunction or cholinergic deficit, neuroinflammation, and dysregulated mitochondrial function have been recognized as prominent features of AD pathology [3,4,5,6,7].

Relevant to the amyloid cascade hypothesis, β-site amyloid precursor protein cleaving enzyme-1 (BACE1, also known as β-secretase or memapsin 2) is an enzyme primarily known for its role in the amyloidogenic pathway leading to the production of Aβ peptides, which are implicated in the pathogenesis of AD [8,9]. BACE1 is a transmembrane aspartic protease type of enzyme that cleaves the amyloid precursor protein (*APP*) at the β-secretase site. This cleavage event generates a soluble fragment called β-C-terminal fragment (β-CTF), which is further processed by γ-secretase to produce Aβ peptides [10]. BACE1-mediated cleavage of APP is considered the rate-limiting step in the production of Aβ peptides. Accumulation of Aβ peptides, particularly Aβ_42_, is a hallmark feature of AD. Aβ peptides aggregate to form insoluble plaques, which are neurotoxic and contribute to synaptic dysfunction, neuronal loss, and cognitive decline in AD [11]. BACE1 activity is, therefore, implicated in the pathogenesis of AD, and inhibition of BACE1 is considered a potential therapeutic strategy for the treatment of the disease. BACE1 is widely expressed in various tissues and cell types, with particularly high expression levels in the brain. It is predominantly localized to the endosomes, Golgi apparatus, and synaptic vesicles within neurons [12]. BACE1 activity is regulated by factors such as subcellular localization, post-translational modifications, and interactions with other proteins. Given its central role in Aβ production and AD pathology, BACE1 has emerged as a promising therapeutic target for the treatment of AD [13]. Efforts to develop BACE1 inhibitors as potential disease-modifying drugs for AD have been ongoing [14], although there has been a relatively limited number of studies on the BACE1 inhibitory effect of natural products. Thus, several selected phenolic compounds found in medicinal plants, i.e., rosmarinic acid, gallic acid, epigallocatechin gallate (EGCG), oleuropein, 3-hydroxytyrosol, and quercetin, were tested against BACE1 for their inhibitory potential. They were also subjected to SH-SY5Y, a human neuroblastoma cell line. Additionally, their effect on levels of fourteen AD-related genes including *APP*, presenilin-1 (*PSEN1*), ATP-binding cassette subfamily A (*ABCA7*), apolipoprotein E (*APOE*), clusterin (*CLU*), phosphatidylinositol binding clathrin assembly protein (*PICALM*), bridging integrator 1 (*BIN1*), CD-associated protein (*CD2AP*), complement C3b/C4b receptor 1 (*CR1*), the cluster of differentiation 33 (*CD33*), sortilin-related receptor 1 (*SORL1*), matrix metalloproteinase 9 (MMP9), tumor necrosis factor-α (*TNF-α*), and C-C motif chemokine ligand 5 (*CCL5*) was analyzed using the real-time polymerase chain reaction (RT-PCR) method, normalized with β-actin.

## 2. Results

### 2.1. BACE1 Inhibition

Percentage inhibition and IC_50_ values of the BACE1 inhibitory effects of the tested phenolic compounds at 10 mM stock concentration are given in Table 1. Compared to quercetin (IC_50_: 3.16 ± 0.30 mM) as the reference as well as the test compound, the most effective compound was EGCG with an IC_50_ value of 1.62 ± 0.12 mM, followed by rosmarinic acid (IC_50_: 4.06 ± 0.68 mM). Oleuropein possessed a low level of inhibition (IC_50_: 9.87 ± 1.01 mM), whereas gallic acid and 3-hydroxytyrosol had BACE1 inhibition below 50%. 

### 2.2. Molecular Docking Studies with BACE1

**Figure 1:**
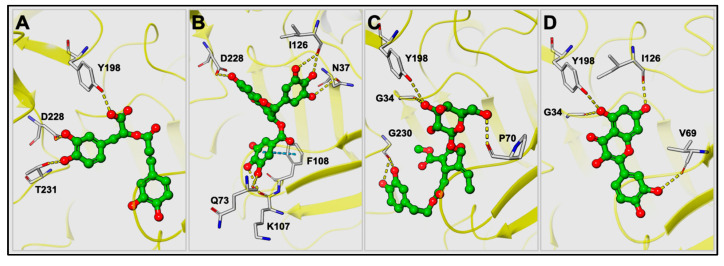
The predicted binding modes for rosmarinic acid (**A**), EGCG (**B**), oleuropein (**C**), and quercetin (**D**) in human BACE1 active site (PDB: 2WJO). The yellow dotted lines represent H-bonds, and the cyan-dotted line represents π-π interactions.

Molecular docking studies were performed to gain better understanding of the molecular interactions of rosmarinic acid, EGCG, oleuropein, and quercetin to the BACE1. The results are presented in Figure 1 and tabulated in Table 2. The results revealed that rosmarinic acid bound into the active site through H-bonding formed between the hydroxyl groups of the benzene ring close to the carboxylic acid moiety and the catalytic aspartic residue D228 and T231. In addition, carboxylic acid oxygen established H-bonding with Y198. EGCG was observed to form six H-bonds between (i) the hydroxyl group of the benzopyran ring and the catalytic aspartic residue D228; (ii) the hydroxyl groups of the trihydroxyphenyl ring attached directly to the benzopyran ring and N37, I126; (iii) the hydroxyl groups of gallate moiety and Q73 and K107. Moreover, the gallate moiety of EGCG was involved in the π-π interactions with F108. The H-bonding interactions between hydroxyl groups and the G23, P70, Y198, and G230 residues were present in the oleuropein:BACE1 complex. Additionally, quercetin was observed to occupy a similar binding site with oleuropein and interacted with the G34, V69, I126, and Y198 residues through H-bonding. 

### 2.3. Cytotoxic Activity Findings Obtained by the Alamar Blue Method

The possible cytotoxic activity of quercetin, rosmarinic acid, EGCG, oleuropein, and gallic acid determined to be most active in terms of the anti-Alzheimer effect was evaluated in SH-SY5Y human neuroblastoma cells by the Alamar Blue method. DMSO was used as solvent control, and cell viability was recorded as 100%. The concentration at which 8% inhibition was observed (IC_08_), i.e., the doses at which 92% viability was observed, were calculated and are given in Table 3.

### 2.4. Expression Analysis Data by RT-PCR Method

**Figure 2:**
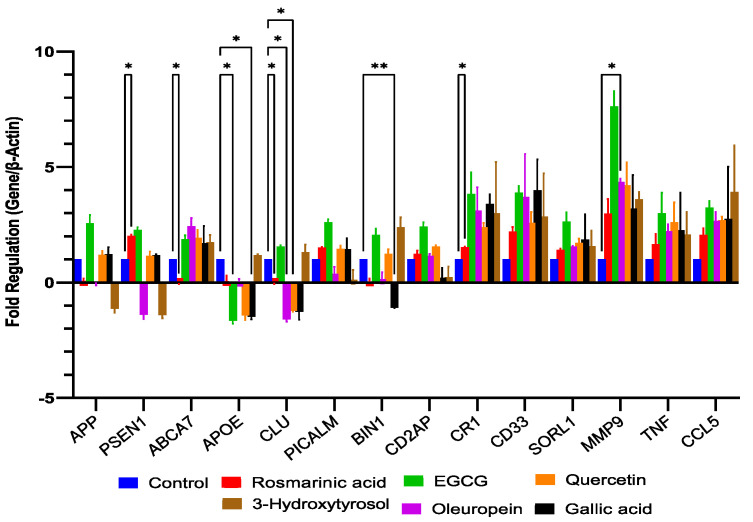
Fold regulation values of rosmarinic acid, EGCG, oleuropein, quercetin, 3–hydroxytyrosol, and gallic acid on genes compared to the control group. Multiple groups were compared by ANOVA and Dunnet *post hoc* test. * *p* < 0.05, ** *p* < 0.01.

PCR results obtained from SH-SY5Y cells treated with rosmarinic acid, gallic acid, EGCG, oleuropein, 3-hydroxytyrosol, and quercetin, tabulated as their fold regulation values, are given in Table 4a,b and Figure 2.

## 3. Discussion

BACE1 is a key enzyme involved in the production of Aβ peptides, which are implicated in the pathogenesis of AD. Nevertheless, quite a limited number of natural products against BACE1 inhibition are available in the literature [15]. Several studies have reported that quercetin exhibits inhibitory effects against BACE1 activity. Quercetin (3,3′,4′,5,7-pentahydroxyflavone) is a common flavonoid derivative with marked biological activities [16,17]. It has been shown to inhibit BACE1-mediated cleavage of APP, leading to reduced production of Aβ peptides. The inhibition of BACE1 activity suggests that quercetin may give a pre-clinical clue for potential therapeutic value in AD by reducing the accumulation of Aβ plaques in the brain. Moreover, Zhumanova et al. provided a statement on the significant effectiveness of five *O*-methylated quercetin derivatives in inhibiting β-secretase with IC_50_ values ranging from 1.2 to 6.5 μM [18]. On the other hand, quercetin induced the expression of *ADAM10* and *ADAM17* genes, which encode proteins involved in the proteolytic shedding of cell surface proteins, including *APP*, thereby exerting its effects [19]. 

Gallic acid (3,4,5-trihydroxybenzoic acid) is a hydroxybenzoic derivative of non-flavonoids classified among polyphenols [20,21]. Quercetin and gallic acid, two common plant phenolics, are known as antioxidants and have displayed neuroprotective action [21,22,23]. It has been shown that the number of regulatory T cells increases, and an anti-inflammatory effect was shown in the presence of gallic acid in the allograft model [22]. Supporting our results, the expression of *TNF-α* is increased after gallic acid treatment through necroptosis [23,24]. In the results, innate immunity and phagocytic receptors related to *CD33* and *CR1* expression and synaptic activity associated with MMP9 expression were observed to increase after gallic acid treatment [25,26]. There was expected to be an increase in other innate immunity-related genes like *ABCA7* and *CLU*; however, there has been no significant change in the expression of these genes. It is also clear that the expression levels of other AD and lipoprotein-related genes revealed by genome-wide association studies (GWAS), such as *APOE*, *PICALM*, *BIN1*, and *CD2AP*, were not changed [27]. Mori et al. reported that GA treatment for 6 months at 20 mg/kg (body weight, b.w.) totally reversed spatial reference learning and memory impairment in *APP/PS1* transgenic mice [28]. Furthermore, cerebral amyloidosis, including brain parenchyma and cerebral vascular β-amyloid deposits, was attenuated, while cerebral Aβ-proteins were reduced in *APP/PS1* mice treated with gallic acid. Consistent with our data, the same study found that although gallic acid directly inhibited BACE1 activity, it did not alter *ADAM10* or BACE1 transcription.

As another strategy for the treatment of AD, the neuroprotective effect of rosmarinic acid through various mechanisms such as cholinesterase inhibitory activity, anti-amyloidogenic effect, etc., has been reported by multiple researchers, including our group [29,30,31,32]. However, the BACE1 inhibitory potential of rosmarinic acid has been described up to date in only one quite earlier study with an IC_50_ value of 0.021 mM [33], which is in accordance with our data. Although rosmarinic acid possessed an IC_50_ value close to that of quercetin as the reference compound, its BACE1 inhibitory effect was weaker than quercetin.

As one of the bioactive green tea polyphenols, EGCG earlier emerged as an attractive polyphenol for an anti-amyloidogenic therapeutic strategy [34]. It was reported to inhibit Aβ_1-42_-induced memory dysfunction and to diminish brain β- and γ-secretase activities [35]. EGCG also blocked lipopolysaccharide (LPS)-induced rise of the Aβ level via the weakening of LPS-induced β- and γ-secretase activities in mice.

According to the literature, 3-hydroxytyrosol has been tested in a pure form only once so far. Hydroxytyrosol (IC_50_: 0.035 ± 0.04 μM) and the hydroxytyrosol-rich extract from olive leaf (each 100 mg of olive leaf extract capsule reported to provide 25 mg of hydroxytyrosol) (IC_50_: 0.26 ± 0.05 μM) was previously reported to have an inhibitory effect against this enzyme, where the reference compound was EGCG (IC_50_: 96.26 μM) [36]. In the same study, oleuropein was identified with an IC_50_ value of (IC_50_: 2.76 ± 0.23 μM). However, the combination of flavonoids (e.g., quercetin, rutin, luteolin, and verbascoside) or non-flavonoids, including the biophenols including hydroxytyrosol and oleuropein, in the extract was concluded to display a synergistic effect. The variation in inhibition levels of 3-hydroxytyrosol against BACE1 observed in different studies could be attributed to several factors, such as assay methods, substrate concentrations, reaction times, and temperature, which can influence the observed inhibition levels. Different studies may employ different protocols, leading to variations in results. Consistently, the exposure of an olive leaf fraction containing oleuropein, hydroxytyrosol, verbascoside, luteolin, and quercetin with the human neuroblastoma SH-SY5Y cell line induced by the neurotoxic agent Aβ_1–42_ led to inverting the loss of viability [36]. 

Some of the previous studies revealed that oleuropein and 3-hydroxytyrosol function to decrease the expression of monocytic inflammatory cytokines and pro-inflammatory cytokine production, including *TNF-α*, *IL-1β*, and nitric oxide [37,38,39], while both compounds may have a neuroprotective effect in addition to their anticancer, antimicrobial, antiviral, and anti-angiogenic effects. It is known that oleuropein can non-covalently bind to Aβ peptide to inhibit its polymerization and fibril formation; therefore, it could be considered a neuroprotective polyphenol [38,40,41]. Similarly, EGCG also has anti-inflammatory and neuroprotective properties, and it has been proven that the production of *TNF-α*-induced monocytes is inhibited after EGCG treatment [42,43,44]. Regarding EGCG’s effect on AD, it shows a neuroprotective effect on cellular and animal models of the disease because it is involved in the Aβ production regulation. EGCG is one of the active components of green tea, and it has also been known that green tea consumption decreases the cognitive impairment risk [15,43]. According to our results, quercetin, oleuropein, EGCG, and 3-hydroxytyrosol have similar patterns in the alteration of gene expression levels of the 14 genes mentioned. The expression levels of synaptic activity related to *MMP9*, immune-related *TNF-α*, *CCL5*, *CD33*, and *CR1* were increased even though direct Aβ-related gene expression did not change significantly. 

Although *NF-κB*-dependent neuroprotective effect has been reported for rosmarinic acid in the literature [45,46,47], no significant effect was observed in this study. The observed outcome could potentially be attributed to the utilization of non-toxic and minimal quantities in the present investigation.

Limited research is available on the effects of these compounds on expressions of *CCL5*, which is involved in immune cell recruitment and neuroinflammation, as well as *SORL1*, which is related to Aβ metabolism and transport. Quercetin and oleuropein have been investigated for their potential to modulate MMP9 expression, which is involved in blood–brain barrier integrity and extracellular matrix remodeling. Quercetin and EGCG have been studied for their effects on *CD3*3 expression, implicated in microglial activation and neuroinflammation.

On the other hand, copper ions (Cu^2+^/Cu^+^) bound to Aβ_42_ in AD undergo redox cycling, leading to the generation of reactive oxygen species (ROS) such as superoxide (O_2_^−^), hydrogen peroxide (H_2_O_2_), and hydroxyl radicals (•OH). These ROS contribute to oxidative stress, neuronal damage, and the progression of AD. The copper-bound Aβ_42_ further exacerbates amyloid plaque formation, creating a feedback loop that amplifies neurodegeneration [48,49]. The phenolic compounds studied herein are well-known for their antioxidant properties, meaning they can scavenge free radicals like superoxide, hydroxyl radicals, and hydrogen peroxide generated by copper-Aβ complexes. Importantly, the protective role of the six phenolic compounds in this work against neurodegenerative diseases, including AD, was demonstrated through their ability to chelate metals such as copper and reduce oxidative stress caused by metal-Aβ complexes [50,51,52]. 

## 4. Materials and Methods

### 4.1. Chemicals

The references of phenolic compounds tested in this study (Table 1) with over 95% purity were acquired from Sigma-Aldrich (St. Louis, MO, USA). 

### 4.2. Beta Secretase (BACE-1) Inhibition Assay

Fluorescence resonance energy transfer (FRET) technology was used to measure the enzymatic activity of BACE1, and measurements were performed by modifying previously used methods and the protocol included in the manufacturer’s (PanVera^®^ Corporation, Madison, MI, USA) assay kit [53]. The tested compounds, human recombinant BACE1 (1.0 U/mL, pH 7.5), buffer (50 mM sodium acetate, pH 4.5), and substrate (Rh-EVNLDAEFK-Quencher, 75 μM in 50 mM ammonium bicarbonate) were added to the reaction wells and incubated at 25 °C for 60 min. The increase in fluorescence intensity produced by substrate hydrolysis was measured on a fluorescence spectrophotometer (SpectraMax i3x microplate reader, Molecular Devices, San Jose, CA, USA) at wavelengths of 545 and 585 nm, respectively. The inhibition rate was obtained using the following equation:

Inhibition% = [1 − (B − B_0_)/(K − K_0_)] × 100

K_0_ = fluorescence value of the control group at minute 0
K = fluorescence value of the control group at 60 min
B_0_ = fluorescence value of the test compound at minute 0
B = fluorescence value of the test compound at 60 min

### 4.3. Molecular Docking Simulations 

The molecular docking studies were carried out using the Glide module implemented in the Schrödinger Small-Molecule Drug Discovery Suite (version 2023-1, Schrödinger, LLC, New York, NY, USA, 2023). The compounds, which were built via the builder panel in Maestro, were subjected to ligand preparation by LigPrep (Schrödinger Release 2023-1: LigPrep, Schrödinger, LLC, New York, NY, USA, 2023) under default conditions. The X-ray crystal structure of human BACE1 [Protein data bank (PDB): 2WJO] was retrieved from the PDB [54]. The protein was prepared using the Protein Preparation Wizard tool. Water molecules were deleted, and hydrogen atoms were added, followed by the assignment of all-atom charges and atom types. Finally, the energy minimization and refinement of the structure were performed up to 0.3 Å RMSD by applying the OPLS4 force field. The centroid of the X-ray ligand was defined as the grid box. Van der Waals (vdW) radius scaling factor 0.80, partial charge cutoff 0.15, and optimized potentials for liquid simulations (OPLS4) force field were used for receptor grid generation. The docking protocol was justified by redocking the co-crystallized ligand, demonstrating a 0.8427 Å root mean square deviation (RMSD) aligned to its bioactive conformation in the crystal structure of BACE1. Next, the compounds prepared by LigPrep were docked into BACE1 using the extra-precision (XP) docking mode of the Glide with a 0.80 vdW radius scaling factor and 0.20 partial charge cutoff [55]. The protocol facilitates docking by ligand flexibility and the generation of multiple conformers within the rigid receptor. The compounds that were docked most favorably were ranked based on the XP scoring protocol.

### 4.4. Cell Culture Experiments

#### Cell Culture

SH-SY5Y, a human neuroblastoma cell line (purchased from ATCC^®^, Cat. No. CRL-2266™), was maintained in Dulbecco’s Modified Eagle Medium/Nutrient Mixture F-12 (DMEM/F-12) complemented with 10% heat-inactivated fetal bovine serum (FBS) and 1% penicillin–streptomycin after being processed for 30 min at 56 °C. Cells were cultivated in a sterile incubator at 37 °C with 5% CO_2_ and 95% relative humidity.

### 4.5. Determination of Cytotoxicity by Alamar Blue Method

In order to determine the possible cytotoxic activity of the phenolic compounds determined to be the most active in terms of the anti-Alzheimer effect in human neuroblastoma cells, their cytotoxic effect on cell viability using SH-SY5Y human neuroblastoma cell line was evaluated using the Alamar Blue method, a fluorometric test method [56]. Cells were maintained in Dulbecco’s Modified Eagle’s Medium (DMEM)/nutrient mixture F-12 (DMEM/F-12) containing 1% penicillin–streptomycin supplemented with 10% fetal bovine serum (FBS) heat-inactivated at 56 °C for 30 min. Cells were cultured in a sterile incubator at 37 °C with 5% CO_2_ and 95% humidity. Then, 10,000 SH-SY5Y cells in 100 µL of complete medium were seeded in each well of a 96-well plate and incubated for 16–24 h for surface attachment. Cells were treated with different concentrations (0–400 µM) of the tested phenolic compounds with BACE1 inhibitory effect (e.g., rosmarinic acid, EGCG, oleuropein, and quercetin) dissolved in DMSO and incubated with 5% CO_2_ at 37 °C for 24 h. Then, 10 µL of filter-sterilized 0.015% resazurin solution (Sigma-Aldrich, R7017) was added to each well and incubated for 2 h at 37 °C with 5% CO_2_. Excitation at 560 nm wavelength and emission fluorescence at 590 nm wavelength were measured, and cell viability% was calculated compared to the control group [57].

### 4.6. Expression Analysis of Selected Phenolic Compounds on Some Genes Associated with AD by Real-Time Polymerase Chain Reaction (Real-Time/RT-PCR) Method

#### RNA Isolation and Real-Time/RT-PCR

SH-SY5Y cells were treated with rosmarinic acid, gallic acid, EGCG, oleuropein, 3-hydroxytyrosol, and quercetin for 24 h to determine their IC_08_ values, and RNA isolation was performed. In the RNA isolation protocol, cell pellets were homogenized using 1 mL of TRIzol reagent and incubated for 5 min at room temperature. Chloroform (200 µL) was added, and the mixture was shaken vigorously and incubated for 3 min at room temperature. The mixture was then centrifuged at 10,000 rpm for 15 min at +4 °C, and the upper colorless phase was transferred to an RNase-free microcentrifuge tube. Isopropanol (500 µL) was added to the tube, and the mixture was incubated at room temperature for 10 min and centrifuged at 10,000 rpm for 10 min. The supernatant was removed, and the pellet was washed with at least 75% ethanol (1 mL) and centrifuged at 7500 rpm for 5 min at +4 °C. After removing all of the supernatant from the tube and air-drying the pellet, the RNA-containing pellet was dissolved in approximately 100 µL of nuclease-free water and incubated at 55 °C for 5 min. RNA was stored at −80 °C for long-term storage.

cDNA synthesis was performed using the ABM, OneScript^®^ Plus cDNA synthesis kit (Applied Biological Materials Inc. (abm), Richmond, BC, Canada). A mixture of RNA (2000 ng/µL), dNTPs (1 µL), oligo (dT) primer (1 µL), oligo (dT) primer (1 µL), and up to 13 µL of nuclease-free water was prepared and incubated at 65 °C for 5 min as described in the protocol supplied with the kit. After incubating the mixture on ice for 1 min, 4 µL of 5X RT buffer and 1 µL of OneScript^®^ Plus RTase were added to the tube. The mixture was incubated at 50 °C for 50 min and 85 °C for 5 min. cDNA was stored at −20 °C for long-term storage.

Real-time PCR was performed using ABM, BlasTaq™ 2X qPCR MasterMix, and the CFX Connect real-time system. The change in gene expression levels of a total of 14 genes (*APP*, *PSEN1*, *ABCA7*, *APOE*, *CLU*, *PICALM*, *BIN1*, *CD2AP*, *CR1*, *CD33*, *SORL1*, *MMP9*, *TNF*, and *CCL5*) is presented in Table 5, which were normalized to β-actin [58]. DMSO, the solvent for dissolving the phenolic compounds, was used as the control group.

### 4.7. Statistical Analysis

The Qiagen GeneGlobe Complimentary qPCR Data Analysis Portal (Redwood City, CA, USA), utilizing default parameters, was employed to calculate changes in gene expression. CT values were exported from the BioRad CFX Connect real-time qPCR system and normalized against a reference gene. The *p*-value was derived from a Student’s *t*-test applied to the replicate 2^−ΔΔCT^ values for each gene in the comparisons between the control and test groups. The *p*-value calculation employed was derived from a parametric, unpaired, two-sample equal variance, two-tailed distribution, a method that is widely recognized in the scientific literature. Fold change calculations, representing gene expression ratios, were determined using the established ΔΔCT method as initially described by Livak and Schmittgen [59]. All other data were presented as Mean ± SD and analyzed using GraphPad Prism 10.0.1 for Windows. The intergroup comparison utilized a *t*-test, with significance determined at a *p*-value threshold of less than 0.05. Multiple groups were compared by ANOVA and Dunnet post hoc test.

## 5. Conclusions

EGCG’s ability to inhibit BACE1 suggests a potential role in mitigating Aβ production and, thus, could be explored further as a therapeutic agent for AD. The remarkable inhibitory effect of rosmarinic acid on BACE1 suggests potential but may require further optimization or investigation in combination with other compounds for enhanced efficacy. While oleuropein exhibited some inhibitory activity against BACE1, its potency was comparatively weaker than EGCG and rosmarinic acid. While gallic acid and 3-hydroxytyrosol may have other benefits for neuroprotective mechanisms, their low inhibition of BACE1 suggests that they may not be as effective in targeting Aβ production. Overall, while there is substantial evidence supporting the potential of quercetin, gallic acid, 3-hydroxytyrosol, oleuropein, rosmarinic acid, and EGCG in modulating various genes and pathways relevant to AD, further research is needed to fully elucidate their therapeutic potential through other mechanisms of action.

Overall, these results highlight the potential of certain phenolic compounds, particularly quercetin, EGCG, and rosmarinic acid, as promising BACE1 inhibitors. Since natural molecules are inspiring precursor compounds for new drug candidates, elucidation of their previously unstudied pharmacological effects is important for drug research. However, further studies, including in vivo efficacy and safety assessments, would be needed to validate their relevant therapeutic potential. Challenges such as bioavailability, pharmacokinetics, and off-target effects need to be addressed before they can be considered viable novel drug candidates for AD.

## Figures and Tables

**Table 1:** BACE-1 inhibitory effects of selected phenolic compounds at 10 mM stock concentration.

Tested Compounds	BACE1 Inhibition (Inhibition% ± SD ^a^)	IC_50_ ± SD ^a^ (mM)
Rosmarinic acid	67.04 ± 0.64	4.06 ± 0.68
Gallic acid	27.51 ± 2.97	- ^b^
Oleuropein	52.38 ± 2.08	9.87 ± 1.01
EGCG	90.58 ± 1.07	1.62 ± 0.12
3-Hydroxytyrosol	45.33 ± 0.22	>10
Quercetin ^c^	86.87 ± 4.48	3.16 ± 0.30

^a^ Standard deviation (n: 3), ^b^ Not calculated due to inhibition below 50%, ^c^ Reference.

**Table 2:** Molecular interactions observed between the docked compounds and BACE1.

Tested Compounds	Binding Energy (XP Gscore, kcal/mol)	H-Bonding	π-π Interaction
Rosmarinic acid	−7.49	Y198, D228, T231	-
EGCG	−10.23	N37, Q73, K107, I126, D228	F108
Oleuropein	−9.59	G34, P70, Y198, G230	-
Quercetin	−9.04	G34, V69, I126, Y198	-

**Table 3:** IC_08_ values of cytotoxicity evaluation of phenolic compounds exposed to SH-SY5Y cells. The results represent the Mean ± SD of two independent replicates from triplicate measurements.

Phenolic Compounds	IC_08_ Values (µM)
Rosmarinic acid	546 ± 31.55
Gallic acid	187 ± 7.11
Oleuropein	171 ± 22.01
EGCG	103 ±12.47
3-Hydroxytyrosol	278 ± 12.21
Quercetin	27 ± 5.30

**Table 4:** (**a**) Fold regulation values of rosmarinic acid, EGCG, and oleuropein on genes compared to the control group. (**b**) Fold regulation values of quercetin, 3-hydroxytyrosol, and gallic acid on genes compared to the control group. Multiple groups were compared by ANOVA and Dunnet *post hoc* test.

**(a)**							
**No.**	**Genes**	**Rosmarinic Acid**		**EGCG**		**Oleuropein**	
		**Fold Regulation**	***p*** **Value**	**Fold Regulation**	***p*** **Value**	**Fold Regulation**	***p*** **Value**
1	*APP*	1.01	0.987519	**2.45** ^a^	**0.09315**	1.05	0.909799
2	*PSEN1*	1.55	**0.027961**	1.75	**0.016982**	−1.07	0.605031
3	*ABCA7*	−1.07	0.744866	1.60	**0.028730**	**2.42**	**0.000844**
4	*APOE*	1.45	0.201926	1.02	0.849047	−1.33	0.2225886
5	*CLU*	1.11	0.494337	1.74	**0.001902**	−1.78	**0.004846**
6	*PICALM*	1.32	0.88009	**2.28**	**0.000377**	1.43	0.055542
7	*BIN1*	1.33	0.1070883	**2.65**	**0.000730**	−1.14	0.435954
8	*CD2AP*	1.08	0.595778	**2.12**	**0.001512**	1.31	**0.032858**
9	*CR1*	1.34	0.261793	**3.02**	**0.010446**	**3.45**	**0.000003**
10	*CD33*	1.88	**0.033980**	**3.47**	**0.008747**	**3.88**	**0.000002**
11	*SORL1*	1.23	0.357220	**2.24**	**0.015080**	1.90	**0.003181**
12	*MMP9*	**3.02**	**0.000025**	**7.86**	**0.000006**	**5.31**	**0.000001**
13	*TNF*	1.75	0.099466	**3.10**	**0.001685**	**2.09**	**0.047554**
14	*CCL5*	1.82	**0.042974**	**3.10**	**0.000600**	**2.67**	**0.002246**
15	*BACT*	1.00	Nan	1.00	Nan	1.00	Nan
(**b**)							
**No.**	**Genes**	**Quercetin**		**3-Hydroxytyrosol**		**Gallic acid**	
		**Fold regulation**	***p*** **Value**	**Fold Regulation**	***p*** **Value**	**Fold Regulation**	***p*** **Value**
1	*APP*	1.14	0.486887	−1.07	0.531044	1.15	0.365962
2	*PSEN1*	1.13	0.3988239	−1.08	0.565985	−1.11	0.459730
3	*ABCA7*	1.82	**0.003884** ^a^	1.82	**0.0028886**	1.55	0.083223
4	*APOE*	−1.02	0.721477	−1.17	0.419436	−1.07	0.64335
5	*CLU*	−1.09	0.427842	1.15	0.352617	−1.11	0.437737
6	*PICALM*	1.26	0.16215	1.21	0.263041	1.22	0.259037
7	*BIN1*	1.58	**0.022014**	1.87	**0.000833**	1.16	0.427674
8	*CD2AP*	1.36	**0.044197**	1.36	0.056017	1.15	0.351825
9	*CR1*	**2.20**	**0.04126**	**2.86**	**0.000826**	**2.20**	**0.025013**
10	*CD33*	**2.29**	**0.006086**	**2.88**	**0.000687**	**2.40**	**0.020188**
11	*SORL1*	1.49	0.075158	1.81	**0.008297**	1.65	0.049611
12	*MMP9*	**4.31**	**0.000006**	**4.38**	**0.000010**	**3.14**	**0.003766**
13	*TNF*	**2.59**	**0.008301**	**2.04**	**0.030457**	**2.06**	**0.049659**
14	*CCL5*	**2.38**	**0.007989**	**3.61**	**0.000005**	1.92	**0.033933**
15	*BACT*	1.00	Nan	1.00	Nan	1.00	Nan

^a^ Statistically varying values are shown in bold.

**Table 5:** Gene-specific primer sequences and primer adhesion temperatures.

Genes	Advance Sequence	Back Sequence	Primer Adhesion Temperature (Annealing) (°C)
*APP*	*GCCCTGCGGAATTGACAAG*	*CCATCTGCATAGTCTGTGTCTG*	62
*PSEN1*	*GCAGTATCCTCGCTGGTGAAGA*	*CAGGCTATGGTTGTGTTCCAGTC*	54.5
*ABCA7*	*CACTCTTCCGAGAGCTAGACAC*	*CTCCATATCTGTGTCCGCAGCA*	54.5
*APOE*	*GGGTCGCTTTTGGGATTACCTG*	*CAACTCCTTCATGGTCTCGTCC*	54.5
*CLU*	*TGCGGATGAAGGACCAGTGTGA*	*TTTCCTGGTCAACCTCTCAGCG*	54.5
*PICALM*	*GGCAGCATTAGAGGAAGAACAGG*	*CTGCTGAGGTGGATACAGGAGA*	54.5
*BIN1*	*CGTCAACACGTTCCAGAGCATC*	*CTTGACCGTGAAGGTGTTGCTC*	54.5
*CD2AP*	*CCAAAGCCTGAACTGATAGCTGC*	*GGACTTGTGGAGCTGCTGGTTT*	54.5
*CR1*	*TAGGTGTCAGCCTGGCTTTGTC*	*GACATCTGGAGGTGGCTGACAT*	54.5
*CD33*	*GTGACTACGGAGAGAACCATCC*	*GCTGTAACACCAGCTCCTCCAA*	54.5
*SORL1*	*GAACACCTGTCTTCGCAACCAG*	*TGTCCAGGTCACAGATGGTGGT*	54.5
*MMP9*	*GGGACGCAGACATCGTCATC*	*TCGTCATCGTCGAAATGGGC*	62
*TNF*	*TGGGATCATTGCCCTGTGAG*	*GGTGTCTGAAGGAGGGGGTA*	62
*CCL5*	*CAGTCGTCTTTGTCACCCGA*	*AGAGCAAGCAGAAACAGGCA*	62
*β-Actin*	*GCCGCCAGCTCACCAT*	*GATGCCTCTCTTGCTCTGGG*	59

## Data Availability

Further inquiries can be directed to the corresponding authors.
